# Joint Assessment of Intended and Unintended Effects of Medications: An Example Using Vascular Endothelial Growth Factor Inhibitors for Neovascular Age-Related Macular Degeneration

**DOI:** 10.1155/2009/540431

**Published:** 2010-02-16

**Authors:** Adrian R. Levy, Shelagh Szabo, Andrew Briggs, Andreas Pleil, Alison Davie, Gergana Zlateva, Jonathan Javitt

**Affiliations:** ^1^Department of Community Health and Epidemiology, Dalhousie University, Halifax, NS, Canada B3H 1V7; ^2^Epidemiology, Oxford Outcomes Ltd., Vancouver, Canada V6B 1P1; ^3^Section of Public Health and Health Policy, University of Glasgow, Glasgow G12 8RZ, UK; ^4^Outcomes Research, Pfizer, La Jolla, CA 92121, USA; ^5^Wilmer Eye Institute, Johns Hopkins University, Baltimore, MD 21287, USA

## Abstract

*Objective*. To estimate the net health benefits of pegaptanib and ranibizumab by considering the impact of visual acuity and unintended effects (cardiovascular and hemorrhagic events) on quality-of-life among persons with neovascular age-related macular degeneration. *Methods*. We designed a probabilistic decision-analytic model using published data. It employed 17 visual health states and three for unintended effects. We calculated incremental net health benefits by subtracting the harms of each medication from the benefit using the quality-adjusted life year (QALY). *Results*. In a hypothetical cohort of 1,000 75-year olds with new-onset bilateral age-related macular degeneration followed for ten years, the mean QALYs per patient is 3.7 for usual care, 4.2 for pegaptanib, and 4.3 for ranibizumab. Net benefits decline with increasing baseline rates of unintended effects. *Interpretation*. Net health benefits present a quantitative, potentially useful tool to assist patients and ophthalmologists in balancing the benefits and harms of interventions for age-related macular degeneration.

## 1. Introduction

In response to shifting expectations from the general public, the regulation of medications is undergoing important changes in Canada and abroad including by the United States' Food and Drug Administration and the European Medicines Agency. In this country, Health Canada has introduced the Progressive Licensing Project in order “to develop a modern, integrated approach to the regulation of pharmaceuticals and biologics that can be implemented throughout the lifecycle of these drugs” [1]. One of the underlying principles is that joint evaluation of the risks and benefits of a medication is to occur throughout its lifecycle and thereby extends the current system which focuses regulatory approval on the premarketing phase. There are formidable hurdles to the proposed new system including the development of metrics that quantitatively incorporate both risks and benefits. One method that has been proposed is the use of decision models that capture all relevant intended and unintended effects [2, 3]. One advantage of decision models is that preferences for different health states can be explicitly incorporated into the decision-making process. In this study, we present the joint estimation of risks and benefits among two intravitreal vascular endothelial growth factor (VEGF) inhibitors recently licensed in Canada and other major markets for treating neovascular age-related macular degeneration. 

Age-related macular degeneration is a progressive degeneration of the macula that occurs in up to one-third of persons aged 65 years and over [4]. Up to 15% of sufferers develop the neovascular form in which the development of choroidal neovascularization (growth of abnormal blood vessels), if untreated, progresses to severe central visual loss, macular scarring, and legal blindness [5]. In recent years two intravitreal VEGF inhibitors have been marketed and put in use in Canada and internationally: in 2004, pegaptanib (Macugen), a VEGF165-specific RNA aptamer [6], and in 2006 ranibizumab (Lucentis) [7, 8], a nonspecific pan-VEGF antibody. While both VEGF inhibitors were shown to be better than usual care (no treatment or photodynamic therapy with verteporfin, if clinically indicated) in slowing the progression of visual loss in the registration trials, the magnitude of benefit was greater in trials of ranibizumab (ANCHOR [7, 8]) than in those of pegaptanib (VISION [6]). No head-to-head trials have been published.

VEGF inhibitors have the potential to affect the cardiovascular system, leading to concerns about the possibility of unintended cardiovascular (particularly, arterial thrombotic events) and hemorrhagic adverse drug reactions [9]. The degree of cardiovascular risk due to VEGF inhibitors is likely to depend on a variety of factors including the concentrations in the systemic circulation, half-life, and breadth of activity. Of note, there is evidence that persons with neovascular age-related macular degeneration may already be at elevated risk of cardiovascular disease [10, 11]. 

The objective of this study was to estimate the net health benefits of intravitreal VEGF inhibitors by jointly considering the impact on duration and quality of life of intended and unintended effects among a hypothetical group of patients with bilateral neovascular age-related macular degeneration. We undertook threshold analyses to determine the levels of cardiovascular and hemorrhagic risks at which greater efficacy may be offset by greater risks of unintended effects.

## 2. Methods

We designed a probabilistic decision analytic model [12] in which the incremental net health benefit was calculated by subtracting the net harms in each treatment from the benefit using the quality-adjusted life year (QALY) [13] as a common metric. The target population was adults aged 75 years with new onset neovascular age-related macular degeneration in the second eye. Hypothetical subjects were entered into the model having the distribution of visual acuity reported in the VISION trial [6]. These subjects had a mean visual acuity of 53 ETDRS letters (Snellen chart 20/63) and an estimated mean baseline health-related quality of life utility of 0.72. Baseline characteristics of the trial samples on whom the hypothetical cohort is based are presented in the Appendix. The cycle length (or equivalently, the duration over which an individual remained in the same health state before having the opportunity to transition to another) was one year and the model was run for ten years.

**Figure 1:**
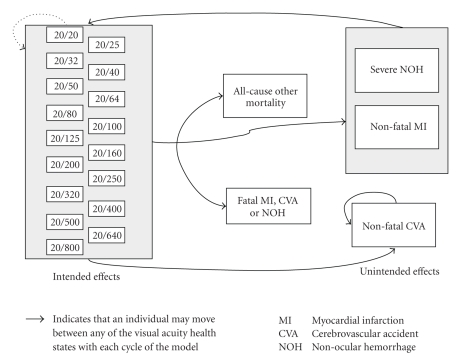
Structure of the risk-benefit decision model.

The Markov model employed 17 health states for classifying the intended effects of treatment, which were based on Early Treatment of Diabetic Retinopathy Study (ETDRS) logMAR visual acuity categories (Figure 1) which ranged from 20/20 (perfect vision) to 20/800 (severe visual impairment; the cutoff for legal blindness is 20/200). During each cycle, subjects could stay in the same state, improve to a better state, or deteriorate to a worse state, and could experience an unintended effect (severe nonocular hemorrhage, nonfatal myocardial infarction (MI), or nonfatal cerebrovascular accident (CVA)), or die (from nonocular hemorrhage, MI, CVA, or from other cause). Transition probabilities for intended and unintended effects were annualized based on the rates reported in the first year of the registration trials for pegaptanib (dose 0.3 mg) or ranibizumab (dose 0.5 mg). Unintended effects from the VISION study of pegaptanib-treated patients [6] were categorized according to the MARINA and ANCHOR study criteria [7, 8]. Subjects who did not die or experience a nonfatal CVA entered the next cycle in the same intended effect health state; it was assumed that subjects recovered fully after experiencing a nonocular hemorrhage or MI. Subjects who experienced a CVA were assumed to remain in that health state until death.

Some proportion of subjects transited to the death state during each cycle, based on all-cause mortality rates from 2002 Canadian life tables [14–16]. As the proportions of fatal MI and CVA in the registration trials were low (<1.0% in all trials; 0.8% among ranibizumab-treated patients (three events), 0.7% among pegaptanib-treated patients (two events), and 0.3% among usual care-treated patients (two events)), these events were accounted for within all-cause mortality rates. The benefits of treatment were measured by the change in visual acuity in the treated eye observed at the end of one year in the registration trials of pegaptanib and ranibizumab (Table 1 and Appendix) [6–8]. Unintended effects of treatment included ranibizumab treatment in the ANCHOR/MARINA trials was associated with relative risks (RR; compared to usual care) of 1.5 (loss of <3 lines of VA), 2.2 (APTC events) and 5.5 (nonocular hemorrhage). Pegaptanib treatment in the VISION Study was associated with RRs (compared to usual care) of 1.2 (loss of <3 lines of VA), 1.5 (APTC events), and 0.8 (nonocular hemorrhage). In the registration trials, the benefits in terms of visual acuity were stronger for ranibizumab than for pegaptanib: 95% of ranibizumab-treated patients experienced less than three lines of ETDRS visual acuity loss, whereas 70% of pegaptanib-treated patients experienced the same outcome.

As the registration trials were of limited duration, an assumption was required about the treatment efficacy after one year. In the base case, the absolute value of the treatment benefit observed in the first year of the trials (intention-to-treat) was assumed to continue without attenuation in the active treatment arms. This assumption was supported by the minimal change observed amongst those who remained on treatment in open-label follow-ups of the VISION (−0.6 letters) [17] and MARINA (−0.6 letters [8]) trials. 

Cardiovascular events (as defined by the Anti-Platelets Trialists' Collaborative (APTC) [18]) and severe nonocular hemorrhage. In threshold analyses designed to simulate populations at differing baseline risk of cardiovascular or nonocular hemorrhagic events, the absolute risk was assumed to increase linearly with baseline risk, while the relative risk of experiencing an event (compared to usual care) remained constant.

Utilities for health states classified by visual acuity levels were obtained from a published study of patients with ophthalmic disease and were based on Snellen visual acuity in the better-seeing eye [19, 20]. Utilities for unintended effects were from patients suffering the event of interest (Table 2) [21–25]. We assumed that MI and NOH events occurred at the beginning of the year, and the utility decrement associated with these events was applied for one model cycle. We assumed the utility decrement associated with CVA to be permanent and apply for the duration of an individual's life (as all trial CVAs were severe). We assumed multiplicative effects on quality of life for persons undergoing more than one unintended health event. This assumption was consistent with the results of another study [26] and avoided the possibility of negative utilities (which would be interpreted as states worse than death, without empirical data). In sensitivity analysis we also examined the effect of using new utility data based on ETDRS (rather than Snellen) visual acuity categories (poster presentation Association for Research in Vision and Ophthalmology 2009 annual meeting) [27]. 

### 2.1. Analyses

QALYs were used as a common metric to capture the joint impact of changes in visual acuity and unintended effects. The incremental net health benefits after ten years were estimated by subtracting the net QALY loss from experiencing unintended effects, from the net QALYs associated with intended effects. All QALYs were discounted at 5% annually to reflect time preference (that costs and benefits that occur in the future are worth less than those currently available) [28]. 

Probabilistic sensitivity analysis was undertaken to examine the robustness of the results by incorporating the joint uncertainty around the estimates of intended and unintended effects and utilities [29]. A beta distribution was applied to randomly generate utility values and adverse event probabilities. The method of moments was used to convert empirically calculated means and standard deviations to parameters of the beta distribution [30]. The Dirichlet distribution was applied to generate fully probabilistic transition matrices for the VEGF inhibitor treatments [29, 30]. Two alternative scenarios were incorporated to examine the impact of different assumptions about the benefits of treatment after 12 months. In the first, the transition probabilities following the first year of treatment remained constant with patients continuing to gain or lose vision at the same rate as during the trial period. Patients therefore continued accruing treatment benefit for the duration of the time horizon of the model. In the second, the treatment benefit was assumed to drop to zero after the first year of treatment and the transition probabilities were those of the usual care group.

Background rates of cardiovascular and hemorrhagic events from the usual care group (Table 1) were incorporated into the base case. To estimate incremental net health benefits in populations at differing underlying risks of unintended effects, threshold analyses were undertaken for populations at differing baseline risks of cardiovascular events and severe nonocular hemorrhage. Increased baseline risks of cardiovascular events up to 2% annual mortality were considered. Increased baseline risks of severe nonocular hemorrhage up to 5% annually were also considered. 

The impact of different discount rates from 0% to 7% [31] was examined in a one-way sensitivity analysis. We also examined five-year and lifetime time horizons. All model results represent the mean values from 1,000 probabilistic simulations. Point estimates of the net health benefit are presented with 95% confidence intervals (95% CI) for the main results. The model was developed in Microsoft Excel 2007 for Windows and is available from the authors upon request.

## 3. Results

**Figure 2:**
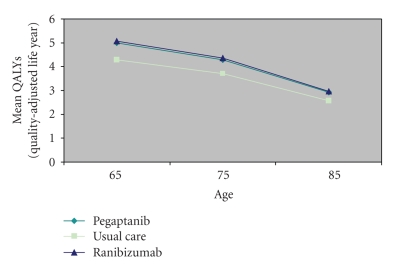
Net health benefits accruing to patients with neovascular age-related macular degeneration when using vascular endothelial growth factor inhibitors according to age (years) at treatment initiation.

In a hypothetical cohort of 1,000 patients with new onset bilateral neovascular age-related macular degeneration followed for ten years, the mean number of QALYs was 3.7 (95% CI, 3.5–4.0) for usual care, 4.3 (95% CI, 4.1–4.4) for pegaptanib, and 4.4 (95% CI, 4.2–4.5) for ranibizumab. Net health benefits declined with increasing age at treatment initiation, from approximately 5.0 (pegaptanib; 4.8–5.2) or 5.1 (ranibizumab; 4.9–5.2) QALYs for patients aged 65 years, to 2.9 (pegaptanib; 2.8–3.0) or 3.0 (ranibizumab; 2.8–3.1) for patients aged 85 years (Figure 2). Net health benefits, and incremental net health benefits, are presented in Table 3.

**Figure 3:**
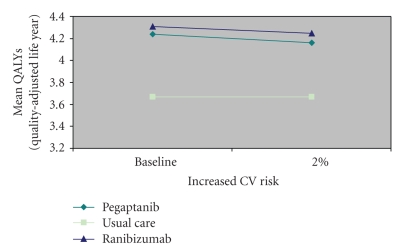
Net health benefits accruing to patients with neovascular age-related macular degeneration when using vascular endothelial growth factor inhibitors according to annual risk of a cardiovascular (CV) event.

**Figure 4:**
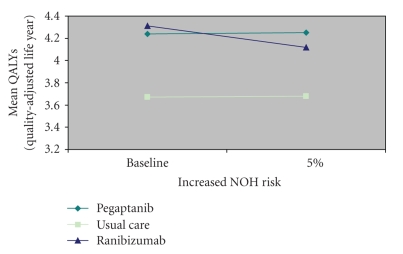
Net health benefits accruing to patients with neovascular age-related macular degeneration when using vascular endothelial growth factor inhibitors according to annual risk of a nonocular hemorrhagic (NOH) event.

As the increased annual risk of cardiovascular events increased up to 2%, the decline in benefit in pegaptanib-treated patients was greater than that seen in ranibizumab-treated patients (to 4.2 versus 4.3 QALYs, resp.; Figure 3). The opposite trend was noted when the annual increased risk of nonocular hemorrhagic events was increased to 5%; the net health benefit declined for ranibizumab-treated patients (to 4.1 QALYs), while the net health benefit for pegaptanib-treated patients remained the same (4.3 QALYs; Figure 4). 

The impact of varying the assumptions regarding duration and magnitude of treatment benefits indicated that ranibizumab proffered the greatest net health benefits under the assumption that all patients continued gaining or losing vision at the same rate beyond the 1-year duration of the trial data (Table 4). Assuming that all treatment benefits dropped to zero after one year of treatment duration resulted in only slightly positive net health benefits among both groups treated with a VEGF inhibitor.

When the model's time horizon was varied, the relative difference between the treatment arms was maintained, although the absolute value of mean QALYs per participant increased with the increasing time horizon of the model. 

When the utility values to quality-adjust life expectancy were varied from those based on Snellen visual acuity categories to ETDRS, the relative difference between the treatment arms was maintained, although the absolute value of mean QALYs per participant increased (Table 5).

## 4. Discussion

Using a decision-analytic framework and data from the literature, we derived a single reproducible metric that captured intended and unintended effects of treatments and incorporated quality of life weights. In this study, we modeled the long-term net health benefits of VEGF inhibitors among patients with bilateral neovascular age-related macular degeneration. Under a realistic assumption that benefits of treatment extended beyond the observed duration of the trials, both pegaptanib and ranibizumab offered positive net health benefits. Based on the results of the registration trials, there is widespread perception among ophthalmologists treating persons with neovascular age-related macular degeneration that ranibizumab is more efficacious than pegaptanib. As in the registration trials, we projected that the absolute benefits were greater in ranibizumab-treated patients. However, when also accounting for unintended effects, the mean difference between the two treatments diminished, to 100 QALYs per 1,000 subjects over a ten year time horizon. There are two potentially nonexclusive reasons for the narrowing of difference: first, the trials of ranibizumab enrolled more patients with predominantly classic subfoveal lesions, the natural history of which are more aggressive. Predominantly classic lesions may therefore appear to respond better to treatment [32, 33], including to VEGF inhibition, than other choroidal neovascularization subtypes. Second, in all three registration trials, approximately three-quarters of subjects had treatment-eye visual acuities upon enrollment of between 20/40 and 20/200, corresponding to utilities between 0.80 and 0.66. Even with apparently large differences in relative benefits in the number of patients experiencing less than three lines of vision loss, the potential gains in quality-adjusted life years were relatively small after incorporating quality of life and risks of unintended effects. The probabilistic net health benefit analyses demonstrated that it is not possible to distinguish statistically between ranibizumab and pegaptanib treatment as measured by QALYs, although the benefits of both active therapies are greater than treatment with usual care.

The analysis suggests that in the case of nonocular hemorrhage as an example, that the net benefit of treatments is dependent on the incidence of the unintended consequence and when the relative rates of unintended consequences change, the net health benefit changes as well.

There are several limitations. First, the validity of the model and results are limited to that of the input data and assumptions. While the intended effects were estimated with precision in the registration trials, only a small number of unintended effects were observed leading to higher uncertainty in these effects. As well, there are theoretical concerns about QALYs that may reduce their validity [34, 35]. Despite those concerns, QALYs have many useful characteristics including that they are straightforward to calculate and interpret and they have been incorporated in many decision models. Second, because of a lack of a head-to-head trial, only indirect comparisons of the two VEGF inhibitors relative to usual care were possible and third, data were extrapolated beyond the duration of the registration trials. Although the VISION study was two years in duration, patients crossed-over after the first year making the year two results challenging to interpret with confidence. We used the first year of the two-year long MARINA trial to make the duration comparable to ANCHOR and VISION. Both these features were addressed by making explicit assumptions and testing the impact of alternative assumptions. Fourth, the source and quality of utility data for intended and unintended effects have a large impact effect on the results and interpretations. For intended effects, we incorporated preference values for health states from a study of 72 patients with macular degeneration of whom only 56 had evidence of neovascular disease. The health states were not standardized and study participants only provided utility estimates for the visual acuity category in which they belonged. The actual numbers of patients from whom mean health state utility estimates were smaller [20, 36] than recommended [37]. Health states were based upon best-corrected Snellen visual acuity which is commonly used to track visual changes in clinical practice. However, Snellen estimates correlate poorly with visual acuities measured on logMAR charts [38], which were used to monitor visual changes in the registration trials [6–8]. As the health states (and utilities) were based on visual acuity in the better-seeing eye we assumed that the better-seeing eye was the treated eye (and that therefore all model patients had developed neovascular disease in their second eye). We therefore incorporated alternate, ETDRS-based preference value estimates as a sensitivity analysis. Fifth, a key component that is poorly understood is the way in which the quality of life and utilities are affected when a person has more than one condition. In this case, we assumed that the utilities were multiplicative, based on another study [26]. Other structures of how utilities combine gave been suggested [39]. Finally, as utilities were only available for visual acuity values for the better-seeing eye, the generalizability of our model was limited to patients with bilateral visual problems where the treated eye measures better visual acuity.

## 5. Conclusion

The framework described here presents one method of addressing Health Canada's Progressive Licensing Project goal of on-going review throughout the lifecycle of new medications: multiple health effects can be incorporated; different sources of data can be accommodated; uncertainty in the input data can be explicitly incorporated; all assumptions are made explicit and the impact of different assumptions can be tested; the time horizon can be extended to include all relevant health effects; and there is a natural link with the model that can be used to assess the economic value proposition.

## Figures and Tables

**Table 1:** Model inputs, intended and unintended effects.

*Intended Effects*	Pegaptanib (*n* = 294)^1^ *n* (%)	Ranibizumab (*n* = 379)^2^ *n* (%)	UC Combined (*n* = 677)^ 3^ *n* (%)
Loss of ≥6 lines ETDRS VA	28 (9.5)	3 (0.8)	118 (39.9)
Loss of ≥3 but <6 lines ETDRS VA	60 (20.4)	15 (4.0)	153 (51.7)
Loss of >0 but <3 lines ETDRS VA	108 (36.7)	83 (21.9)	227 (76.7)
Gain of ≥0 but <3 lines ETDRS VA	80 (27.2)	140 (36.9)	153 (51.7)
Gain of ≥3 lines ETDRS VA	18 (6.1)	138 (36.4)	26 (8.8)

*Unintended Effects*			

Nonfatal MI, % (SE)	0.3 (0.378)	1.1 (0.525)	0.6 (0.295)
RR (95% CI) Nonfatal MI, versus UC	0.5 (0.0–5.5)	2.0 (0.4–10.9)	—
Nonfatal CVA, % (SE)	1.0 (0.586)	1.1 (0.525)	0.4 (0.255)
RR (95% CI) Nonfatal CVA, versus UC	3.0 (0.3–28.9)	2.0 (0.4–10.9)	—
Nonfatal severe NOH, % (SE)	1.0 (0.653)	1.5 (0.614)	0.6 (0.295)
RR (95% CI) Nonfatal severe NOH, versus UC	1.0 (0.2–4.9)	5.5 (0.7–46.3)	—

^1^From VISION Study [6] of 0.3 mg pegaptanib, and VISION-study safety and efficacy summaries provided by Pfizer Inc.

^2^Pooled estimates from MARINA and ANCHOR [7, 8] studies of 0.5 mg ranibizumab.

^3^Pooled estimates from the control arms of the VISION [6], ANCHOR and MARINA [7, 8] studies.

ETDRS VA: Early Treatment of Diabetic Retinopathy Study visual acuity; MI: myocardial infarction; SE-standard error; 95%CI: 95% confidence interval; UC: Usual care; NOH: nonocular hemorrhage.

**Table 2:** Utilities, intended and unintended effects.

	Mean Utility (95% CI)
*Intended Effects* ^1^	
BCVA in the better-seeing eye	

20/20	0.92 (0.87–0.97)
20/25	0.87 (0.82–0.92)
20/32	0.84 (0.79–0.89)
20/40	0.80 (0.74–0.86)
20/50	0.77 (0.70–0.84)
20/63	0.74 (0.67–0.81)
20/80	0.74 (0.67–0.81)
20/100	0.67 (0.57–0.77)
20/125	0.67 (0.57–0.77)
20/160	0.66 (0.55–0.77)
20/200	0.66 (0.55–0.77)
20/250	0.63 (0.54–0.72)
20/320	0.63 (0.54–0.72)
20/400	0.54 (0.43–0.65)
20/500	0.54 (0.43–0.65)
20/640	0.54 (0.43–0.65)
20/800	0.52 (0.36–0.68)
*Unintended Effects*	

CVA^2^	0.52 (0.42–0.62)
MI^3^	0.88 (0.84–0.93)
NOH^4^	0.81 (0.74–0.87)

^1^From Brown et al. [19], originally referenced in Brown 1999 [20].

^2^Calculated based on utility decrement associated with a major stroke, from meta-analysis by Tengs and Lin 2003 [21].

^3^From Tsevat et al. [22].

^4^Based on 3-month utility for a severe gastrointestinal hemorrhage requiring medical intervention, from Maetzel et al. 2001 [23]. Patients were assumed to return to full health for the duration of the cycle.

95% CI: 95% confidence interval; BCVA: best-corrected visual acuity; CVA: cerebrovascular accident; MI: myocardial infarction; NOH: nonocular hemorrhage.

**Table 3:** Net health benefits (with 95% CI), and incremental net health benefits, of VEGF inhibitor treatment in NV-AMD patients, by age at treatment initiation (in quality-adjusted life years).

	Net Health Benefit (95% CI)		
	Age 65 y	Age 75 y	Age 85 y

Ranibizumab	5.06 (4.87–5.23)	4.36 (4.19–4.52)	2.97 (2.85–3.08)
Pegaptanib	4.98 (4.80–5.17)	4.29 (4.14–4.45)	2.93 (2.81–3.03)
UC	4.31 (4.00–4.60)	3.74 (3.48–3.98)	2.59 (2.44–2.75)

	Incremental Net Health Benefit		
	Age 65 y	Age 75 y	Age 85 y

Ranibizumab versus Pegaptanib	0.07 (0.01–0.14)	0.06 (0.01–0.12)	0.04 (0.01–0.08)
Pegaptanib versus UC	0.68 (0.40–0.95)	0.56 (0.34–0.77)	0.34 (0.21–0.47)
Ranibizumab versus UC	0.75 (0.45–1.04)	0.62 (0.38–0.86)	0.38 (0.24–0.53)

95% CI = 95% confidence interval; UC = Usual care; y = years.

Net health benefits were calculated by subtracting the net harms in each treatment from the benefit using the quality-adjusted life year as a common metric.

Incremental net health benefits were calculated by subtracting the net health benefits of two of the treatment arms.

**Table 4:** Mean quality-adjusted life years associated with alternate intended effects scenarios incorporated over the duration of the model.

	Net Health Benefit		
	Base Case	Scenario 1	Scenario 2
Ranibizumab	4.36 (4.19–4.52)	4.51 (4.34–4.69)	3.81 (3.60–4.03)
Pegaptanib	4.29 (4.14–4.45)	4.00 (3.81–4.17)	3.79 (3.57–4.02)

Scenario 1: Transition probabilities following the first year of treatment remained constant, with patients continuing to gain or lose vision at the same rate as during the trial period. Patients therefore continue accruing treatment benefit for the duration of the time horizon of the model.

Scenario 2: Treatment benefit was assumed to drop to zero after the first year of treatment and the transition probabilities were those of the usual care group.

Net health benefits were calculated by subtracting the net harms in each treatment from the benefit using the quality-adjusted life year as a common metric.

**Table 5:** Net health benefits, and incremental net health benefits, in quality-adjusted life years, of VEGF inhibitor treatment in NV-AMD patients, incorporating utility values based on ETDRS visual acuity categories.

	Net Health Benefit		
	Age 65 y	Age 75 y	Age 85 y
Ranibizumab	5.99 (5.90–6.09)	5.15 (5.07–5.23)	3.51 (3.46–3.57)
Pegaptanib	5.89 (5.79–5.99)	5.07 (4.99–5.15)	3.46 (3.40–3.51)
UC	5.26 (5.10–5.41)	4.54 (4.42–4.67)	3.13 (3.05–3.22)

	Incremental Net Health Benefit		
	Age 65 y	Age 75 y	Age 85 y

Ranibizumab versus Pegaptanib	0.09 (0.03–0.16)	0.08 (0.03–0.14)	0.06 (0.02–0.09)
Pegaptanib versus UC	0.64 (0.50–0.77)	0.53 (0.42–0.64)	0.32 (0.25–0.39)
Ranibizumab versus UC	0.73 (0.59–0.87)	0.61 (0.48–0.74)	0.38 (0.30–0.45)

UC = Usual care; y = years.

Net health benefits were calculated by subtracting the net harms in each treatment from the benefit using the quality-adjusted life year as a common metric.

Incremental net health benefits were calculated by subtracting the net health benefits of two of the treatment arms.

**Table 6:** Characteristics of study populations at baseline.

		Pegaptanib^1^ 0.3 mg (*n* = 295)	Pegaptanib^1^ UC (*n* = 296)	Ranibizumab^2^ (*n* = 379)	Ranibizumab^2^ UC (*n* = 381)
Sex, no(%)					
Male		133 (45)	120 (40)	163 (43)	143 (38)
Female		164 (55)	178 (60)	217 (57)	238 (62)

Race, no(%)					
White		283 (96)	284 (95)	268 (97)	371 (97)
Other		12 (4)	14 (5)	12 (3)	10 (3)

Age, no(%)					
50–64		19 (6)	21 (7)	30 (8)	19 (5)
65–74		86 (29)	94 (32)	104 (27)	102 (27)
75–84		155 (53)	160 (54)	188 (50)	206 (54)
≥85		35 (12)	23 (8)	57 (15)	54 (14)
Mean				76.6	77.3
Range				52–93	53–95

LC, no(%)					
PC		72 (24)	76 (26)	135 (36)	141 (37)
MC		111 (38)	102 (34)	96 (25)	89 (23)
OC		112 (38)	120 (40)	149 (39)	151 (40)

Mean LS		3.7	4.2	3.5	3.5
ETDRS VA	Mean	56.2	52.7	51.3	50.6
	≤20/200	45 (15)	45 (15)	63 (17)	78 (20)
	20/40 > VA > 20/200	222 (76)	221 (75)	274 (72)	267 (71)
	≥20/40	27 (9)	30 (10)	42 (11)	39 (9)

^1^From VISION Study [6] of pegaptanib, and safety and efficacy summaries provided by Pfizer Inc.

^2^Pooled estimates from MARINA and ANCHOR [7, 8] studies of ranibizumab.

UC: usual care; LC: lesion composition; PC: predominantly classic; MC: minimally classic; OC: occult with no classic; LS: lesion size; ETDRS VA: early Treatment of Diabetic Retinopathy Study visual acuity.

## Appendix

Input data were abstracted from the results of published randomized trials of active comparator versus usual care [6–8]. Baseline characteristics of the trial groups are presented in Table 6. Usual care in the MARINA Study, examining the safety and efficacy of ranibizumab for NV-AMD patients with OC or MC lesions, was sham treatment [8]; in the ANCHOR Study, focusing on patients with PC lesions, usual care was either PDT with verteporfin at the discretion of the treating investigator or sham therapy [7]. Usual care in the VISION Trial of pegaptanib consisted of sham treatment for patients with OC and MC lesions or PDT with verteporfin at the direction of the treating investigator for those with PC lesions [6]. To allow incorporation of the data into the net benefit decision model, the 0.5 mg treatment groups were combined from ANCHOR and MARINA, and the usual care groups from MARINA, ANCHOR, and VISION were pooled. The treatment effect (relative differences between usual care and treatment in each trial) was maintained by applying the relative risks of experiencing intended and unintended effects to the pooled data, to determine the estimates of intended and unintended effect rates for the pegaptanib and ranibizumab groups.

Intended and unintended effects were considered for the indicated doses only: 0.3 mg for pegaptanib, and 0.5 mg for ranibizumab. Intended effects were quantified using the distribution of changes in visual acuity over one year, with baseline visual acuity distributions for all treatment groups taken from the VISION Study.

## References

[1] Health Canada (2008). What is the progressive licensing project?. *Generic*.

[2] Briggs AH, Levy AR (2006). Pharmacoeconomics and pharmacoepidemiology: curious bedfellows or a match made in heaven?. *PharmacoEconomics*.

[3] Hughes DA, Bayoumi AM, Pirmohamed M (2007). Current assessment of risk-benefit by regulators: is it time to introduce decision analyses?. *Clinical Pharmacology and Therapeutics*.

[4] Klein R, Klein BEK, Linton KLP (1992). Prevalence of age-related maculopathy. The Beaver Dam Eye Study. *Ophthalmology*.

[5] Ferris FL, Fine SL, Hyman L (1984). Age-related macular degeneration and blindness due to neovascular maculopathy. *Archives of Ophthalmology*.

[6] Gragoudas ES, Adamis AP, Cunningham ET, Feinsod M, Guyer DR (2004). Pegaptanib for neovascular age-related macular degeneration. *The New England Journal of Medicine*.

[7] Brown DM, Kaiser PK, Michels M (2006). Ranibizumab versus verteporfin for neovascular age-related macular degeneration. *The New England Journal of Medicine*.

[8] Rosenfeld PJ, Brown DM, Heier JS (2006). Ranibizumab for neovascular age-related macular degeneration. *The New England Journal of Medicine*.

[9] Genentech (January 2007). SAILOR Safety Information—Interim Analysis.

[10] Tan JSL, Wang JJ, Liew G, Rochtchina E, Mitchell P (2008). Age-related macular degeneration and mortality from cardiovascular disease or stroke. *British Journal of Ophthalmology*.

[11] Wong TY, Klein R, Sun C (2006). Age-related macular degeneration and risk for stroke. *Annals of Internal Medicine*.

[12] Glasziou PP, Irwig LM (1995). An evidence based approach to individualising treatment. *British Medical Journal*.

[13] Weinstein MC, Stason WB (1977). Foundations of cost effectiveness analysis for health and medical practices. *The New England Journal of Medicine*.

[14] Statistics Canada Complete life table, Canada, 2000 to 2002, males. http://www.statcan.gc.ca/pub/84-537-x/t/pdf/4198612-eng.pdf.

[15] Statistics Canada Complete life table, Canada, 2000 to 2002, females. http://www.statcan.gc.ca/pub/84-537-x/t/pdf/4198611-eng.pdf.

[16] http://www.statcan.gc.ca/pub/84-537-x/4064441-eng.htm.

[17] Chakravarthy U, Adamis AP, Cunningham ET (2006). Year 2 efficacy results of 2 randomized controlled clinical trials of pegaptanib for neovascular age-related macular degeneration. *Ophthalmology*.

[18] (1994). Collaborative overview of randomised trials of antiplatelet therapy—I: prevention of death, myocardial infarction, and stroke by prolonged antiplatelet therapy in various categories of patients. Antiplatelet Trialists’ Collaboration. *British Medical Journal*.

[19] Brown MM, Brown GC, Stein JD, Roth Z, Campanella J, Beauchamp GR (2005). Age-related macular degeneration: economic burden and value-based medicine analysis. *Canadian Journal of Ophthalmology*.

[20] Brown GC (2000). Vision and quality-of-life. Trans Am Ophthalmol Soc 1999;97:473–511. *American Journal of Ophthalmology*.

[21] Tengs TO, Lin TH (2003). A meta-analysis of quality-of-life estimates for stroke. *PharmacoEconomics*.

[22] Tsevat J, Goldman L, Soukup JR (1993). Stability of time-tradoff utilities in survivors of myocardial infarction. *Medical Decision Making*.

[23] Maetzel A, Hrahn M, Naglie G (2001). The cost-effectiveness of celecoxib and rofecoxib in patients with osteoarthritis or rheumatoid arthritis.

[24] Groeneveld PW, Lieu TA, Fendrick AM (2001). Quality of life measurement clarifies the cost-effectiveness of Helicobacter pylori eradication in peptic ulcer disease and uninvestigated dyspepsia. *American Journal of Gastroenterology*.

[25] National Collaborating Centre for Chronic Conditions (2006). *Hypertension: Management in Adults in Primary Care: Pharmacological Update*.

[26] Flanagan W, McIntosh CN, Le Petit C, Berthelot J-M (2006). Deriving utility scores for co-morbid conditions: a test of the multiplicative model for combining individual condition scores. *Population Health Metrics*.

[27] Pleil AM, Szabo SM, Beusterien KM (2009). Health state classification system for deriving preference weights in neovascular age-related macular degeneration (NV-AMD). *Investigative Ophthalmology & Visual Science*.

[28] Lipscomb J, Weinstein MC, Torrance GW, Gold MR, Siegel JE, Russel LB (1996). Time preference. *Cost-Effectiveness in Health and Medicine*.

[29] Briggs AH, Ades AE, Price MJ (2003). Probabilistic sensitivity analysis for decision trees with multiple branches: use of the Dirichlet distribution in a Bayesian framework. *Medical Decision Making*.

[30] Briggs AH, Sculpher MJ, Claxton K (2006). *Decision Modelling for Health Economic Evaluation*.

[31] ISPOR (2007). *Pharmacoeconomic Guidelines around the World*.

[32] Bressler NM (2001). Photodynamic therapy of subfoveal choroidal neovascularization in age-related macular degeneration with verteporfin: two-year results of 2 randomized clinical trials—tap report 2. *Archives of Ophthalmology*.

[33] Arnold J, Barbezetto I, Birngruber R (2001). Verteporfin therapy of subfoveal choroidal neovascularization in age-related macular degeneration: two-year results of a randomized clinical trial including lesions with occult with no classic choroidal neovascularization—verteporfin in photodynamic therapy report 2. *American Journal of Ophthalmology*.

[34] Mehrez A, Gafni A (1989). Quality-adjusted life years, utility theory, and healthy-years equivalents. *Medical Decision Making*.

[35] Mehrez A, Gafni A (1993). Healthy-years equivalents versus quality-adjusted life years: in pursuit of progress. *Medical Decision Making*.

[36] Brown GC, Sharma S, Brown MM, Kistler J (2000). Utility values and age-related macular degeneration. *Archives of Ophthalmology*.

[37] Furlong W, Feeny D, Torrance GW, Barr R, Horsman J (1990). Guide to design and development of health-state utility instrumentation.

[38] Falkenstein IA, Cochran DE, Azen SP (2008). Comparison of visual acuity in macular degeneration patients measured with snellen and early treatment diabetic retinopathy study charts. *Ophthalmology*.

[39] Fu AZ, Kattan MW (2008). Utilities should not be multiplied: Evidence from preference-based scores in the United States. *Medecal Care*.

